# Prediction of plant promoters based on hexamers and random triplet pair analysis

**DOI:** 10.1186/1748-7188-6-19

**Published:** 2011-06-28

**Authors:** A K M Azad, Saima Shahid, Nasimul Noman, Hyunju Lee

**Affiliations:** 1 Department of Information and Communications, Gwangju Institute of Science and Technology, South Korea; 2 Department of Biochemistry and Molecular Biology, University of Dhaka, Bangladesh; 3 Department of Electrical Engineering & Info Systems, Graduate School of Engineering, University of Tokyo, Japan

## Abstract

**Background:**

With an increasing number of plant genome sequences, it has become important to develop a robust computational method for detecting plant promoters. Although a wide variety of programs are currently available, prediction accuracy of these still requires further improvement. The limitations of these methods can be addressed by selecting appropriate features for distinguishing promoters and non-promoters.

**Methods:**

In this study, we proposed two feature selection approaches based on hexamer sequences: the Frequency Distribution Analyzed Feature Selection Algorithm (FDAFSA) and the Random Triplet Pair Feature Selecting Genetic Algorithm (RTPFSGA). In FDAFSA, adjacent triplet-pairs (hexamer sequences) were selected based on the difference in the frequency of hexamers between promoters and non-promoters. In RTPFSGA, random triplet-pairs (RTPs) were selected by exploiting a genetic algorithm that distinguishes frequencies of non-adjacent triplet pairs between promoters and non-promoters. Then, a support vector machine (SVM), a nonlinear machine-learning algorithm, was used to classify promoters and non-promoters by combining these two feature selection approaches. We referred to this novel algorithm as PromoBot.

**Results:**

Promoter sequences were collected from the PlantProm database. Non-promoter sequences were collected from plant mRNA, rRNA, and tRNA of PlantGDB and plant miRNA of miRBase. Then, in order to validate the proposed algorithm, we applied a 5-fold cross validation test. Training data sets were used to select features based on FDAFSA and RTPFSGA, and these features were used to train the SVM. We achieved 89% sensitivity and 86% specificity.

**Conclusions:**

We compared our PromoBot algorithm to five other algorithms. It was found that the sensitivity and specificity of PromoBot performed well (or even better) with the algorithms tested. These results show that the two proposed feature selection methods based on hexamer frequencies and random triplet-pair could be successfully incorporated into a supervised machine learning method in promoter classification problem. As such, we expect that PromoBot can be used to help identify new plant promoters. Source codes and analysis results of this work could be provided upon request.

## Background

Promoters are non-coding regions in genomic DNA that contain information crucial to the activation or repression of downstream genes. Located upstream of the transcription start site (TSS) of a gene, the promoter region consists of certain short conserved DNA sequences known as cis-elements or motifs, which are recognized and bound by specific transcription factors [1]. Transcriptional regulation of gene expression thus depends on various interactions between these cis-elements and their respective transcription factors.

The accurate identification of promoters and TSS localization remains a major challenge in bioinformatics due to the great degree of diversity observed in the gene and species specific architectures of such regulatory sequences. The first comprehensive review of publicly available promoter prediction tools was made by Fickett and Hatzigeorgiou [2]. However, this program demonstrated a high rate of false positive prediction, mainly because they relied on only one or two given sequence feature characteristics of the promoter region, such as the presence of a TATA box or Initiator element. Ohler [3] then integrated some physical properties of DNA, such as DNA bendability and CpG content, along with the sequence features in their proposed method (referred to as McPromoter), though their approach was developed based on only a particular species, *Drosophila*. And Knudsen [4] developed Promoter 2.0 by combining a neural network and a genetic algorithm that recognized all five promoter sites on a positive strand in a complete Adenovirus genome, but also included 30 false predictions. Another eukaryotic promoter prediction algorithm, TSSW, had 42% accuracy with one false positive per 789 bp [5]. It should also be noted that most of these algorithms were trained exclusively for a specific animal species, and as such their prediction reliability further decreased when applied to distant species, particularly plants.

The first promoter prediction tool trained and adapted for plants was TSSP-TCM, created by Shahmuradov [6]. It used confidence estimation along with a support vector machine (SVM) to predict plant promoters. TSSP-TCM correctly identified 35 out of 40 test TATA promoters and 21 out of 25 TATA-less promoters; the predicted TSSs deviating 5-14 bp from their true positions [6]. However, recent studies have shown that TATA boxes and Initiators are not universal features for characterizing plant promoters, and that other motifs such as Y patches may play a major role in the transcription process in plants [7]. For example, around 50% of rice genes contain Y patches in their promoter regions [8]. However, identification of the true promoter region in long genomic sequences using known regulatory motifs, such as TATA box or Y patch, is extremely difficult due to the short length and degenerative nature of these elements. Hence, prediction methods based on a few known elements may not provide the best results for identifying promoters in plant genomes.

In order to devise a more effective approach for identifying plant promoters, several structural and sequence dependent properties, such as curvature and periodicity in experimentally validated promoters (both TATA-plus and TATA-less types), were analyzed by Pandey [9]. The analysis revealed that the DNA curvature in promoter regions was greater than that in gene containing regions, indicating the possibility of distant sequences being nearer to the core promoter elements and thus affecting regulation of gene expression in the promoter region. To improve the promoter prediction, the use of DNA structural properties such as bendability, B-DNA twist, and duplex-free energy has been further explored for several eukaryotic genomes, including plants [10,11]. And though each of these approaches has shown that a distinct structural profile is associated with core promoter regions, it is still unknown to what extent such DNA-structural properties are related to the presence of known or novel regulatory elements in the plant promoter. Hence, the possibility of distal elements underlying such distinct structural patterns needs to be further explored in order to more fully characterize the actual promoter regions.

In most of the promoter prediction approaches currently available, only protein-coding sequences are used as a non-promoter dataset for training. However, there are other regions in genomic DNA that are neither coding regions nor promoters. For example, miRNA, ribosomal RNA, and tRNA genes are not translated to proteins but have their own promoters. These genes constitute a significant part of the genome that belongs to non-promoter regions. Hence, building a non-promoter dataset that consists of such RNA genes, along with the protein-coding sequences, may improve program efficiency in discriminating between promoter and non-promoter sequences.

Recently, a novel approach (PromMachine) used a characteristic tetramer frequency analysis along with SVM to predict plant promoters [12]. In this approach, all possible tetramer combinations for the nucleotides A, T, G, and C (4^4 ^= 256) were generated. The most significant tetramers (128 in total) were then taken as discriminating features between the promoters and non-promoters. This approach was not dependent on the presence of TATA boxes or Initiator motifs, though it also had several drawbacks. For example, the non-promoter dataset used for training was built only from the protein-coding sequences, with no other non-promoter sequences included, such as non-coding RNA gene sequences. Also, the program could not locate the TSS position when the TATA box was not present [12]. This limits the utility of PromMachine in detecting TSSs for a huge number of plant promoters, as only ~19% of rice genes and 29% of Arabidopsis genes contain TATA box in their core promoters [8,13]. Since the prediction accuracy of PromMachine using 7-fold cross-validation was ~83.91%, the achievement of better accuracy still remains a challenge. As such, the development of a standard validation protocol is important in order to determine the best performing promoter prediction program. To this end Abeel *et al *[14] proposed a set of validation protocols for the fair evaluation of promoter prediction programs aiming to identify a gold standard. Among these protocols, two were based on a binning approach (bins of 500 bp) in which each bin was checked to see whether it overlapped with an experimentally known transcription start region (TSR) or a known start position of a gene. The remaining protocols were based on distance, in which a prediction was considered to be correct if the distance to the closest TSR was smaller than 500 bp. Based on their investigation they proposed a standard for evaluating promoter prediction software, and identified four highly performing software programs; although each of these programs works on different principles and were designed for different tasks [14].

In this study, we proposed two approaches for feature selection that can improve prediction accuracies and analyze the concept of frequently occurring triplet pairs in sequences. The first feature selection approach is the Frequency Distribution Analyzed Feature Selection Algorithm (FDAFSA), in which we counted the frequency of hexamers (adjacent triplet pairs) in a dataset. The second approach is the Random Triplet Pair Feature Selecting Genetic Algorithm (RTPFSGA), where we used the genetic algorithm to find random triplet pairs (RTPs), which randomly pairs two nonadjacent triplets. It should be noted that the distribution of triplet frequencies has been analyzed in many previous studies to identify genes, as the significance of nucleotide triplets that act as codons in coding sequences is universally known. Recent studies have also found that distant amino acids in protein sequences may become adjacent in the tertiary structure and form local spatial patterns (LSP), which may play an important role in the protein's biological functionality [15,16]. Hence, the distribution of triplet frequency may also be useful for identifying promoter regions, as differential patterns of triplet over/under-representation have been discovered in a large number of genomes from diverse species over the last few years [17-19].

These observations support the concept of using RTP as a discriminative feature. In our proposed RTPFSGA, the triplets in each pair are essentially non-adjacent to facilitate the analysis of distant triplets that may become adjacent and act as pairs in three dimensional structures, and to enable identification of significant RTP distributions in coding and non-coding promoter sequences for classification purposes. By combining distinct features selected by FDAFSA and RTPFSGA, and SVM for classification of promoter and non-promoter sequences, we developed PromoBot, as an alternative technique for promoter identification. PromoBot was found to be comparable to, and even outperform, other existing algorithms in classifying plant promoters.

## Methods

### Datasets

Two datasets were used in selecting features and estimating the performance of the promoter classification algorithm: the plant promoter sequence dataset, and the non-promoter sequence dataset.

### Plant promoter sequence database

For this study, 305 experimentally validated plant promoter sequences, collected from the PlantProm database [20], were used as a positive dataset. PlantProm is an annotated, non-redundant collection of proximal promoter sequences for RNA polymerase II from different plant species. In the PlantProm database, all promoter sequences have experimentally verified TSSs [20] and sequence segments are from -200 to +51 bp relative to TSS.

### Non-promoter sequence database

A set of non-redundant plant mRNA, tRNA, and rRNA sequences of various species extracted from PlantGDB [21] as well as miRNA precursor sequences downloaded from miRBase [22] were used to construct the negative dataset. We collected 305 sequences having ≥ 251 bp in length from a list of different plant species (Additional File 1). We had chosen a random start position in each non-promoter sequence and then extracted 251 bp, so that all promoter and non-promoter sequences are of the same length.

### Support vector machine

Support vector machine (SVM) is a supervised machine-learning algorithm that is used to solve classification and regression problems. For binary classifications, candidate input datasets are assumed to be two sets of vectors in an *n*-dimensional space. SVM generates a hyper-plane in the space and uses the maximum margin between these two sets of vectors. Then, two parallel hyper-planes on each side of the separating hyper-plane are constructed to calculate the margin. In this method, a good classification depends on the good separation of spaces, which is accomplished via a hyper plane that ensures a maximum distance to the neighboring data points of both classes [23]. In this study, we used LIBSVM http://www.csie.ntu.edu.tw/~cjlin/libsvm/.

### Feature selection

Success of SVM classification largely depends on the features chosen. In this study, two different approaches were proposed for feature selection: FDAFSA and RTPFSGA. The final version, PromoBot, was built after being trained using the SVM-TRAIN tool of LIBSVM, based on the extracted distinct features from these two feature-selection approaches. In order to use the 5-fold cross validation test, both the promoter and non-promoter datasets were partitioned into 5 groups of promoters and 5 groups of non-promoters; 4 groups were used for selecting features and the remaining group was used for testing. Each set of training data contained 244 promoters and 244 non-promoters, and each test data had 61 promoters and 61 non-promoters.

#### FDAFSA

In PromMachine [12], tetramers were used for the analysis. Here, we used a similar concept in FDAFSA but with hexamers, because we had empirical results that hexamers provided better accuracy than PromMachine's use of tetramers (further discussed in the Results section). In both cases, *training_data_k _*for the k^th ^test in a 5-fold cross validation was used for feature selection and training, and *test_data_k _*was then used for testing. All possible combinations of 'A', 'T', 'C', and 'G' for hexamers were 4,096 (= 4^6^). In FDAFSA, *f_i, j _*and *fn_i, j _*were calculated first where *f_i, j _*was the frequency of i^th ^hexamer in j^th ^known promoter sequence and *fn_i, j _*was the frequency of i^th ^hexamer in j^th ^known non-promoter sequence in *training_data_k_*. We considered both strands of each sequence (plus and minus strands) for hexamer frequency analysis, and then CP_i _and CNP_i _were calculated using Eq. 1 and Eq. 2 respectively.(1)

, where CP_i _was the total frequency of the i^th ^hexamer in all promoter sequences, and *n *was the number of promoters in *training_data_k_*. Next,(2)

, where CNP_i _was the summation of counts in all non-promoter sequences for the i^th ^hexamer, and *n *was the number of non-promoters in *training_data_k_*. The absolute difference between the counts of these 4096 possible hexamers in the known promoter and non-promoter sequences was subsequently calculated for the i^th ^hexamer as follows:(3)

We next sorted hexamers based on Diff_i_, and finally we had *hexamer_set_k_*, which was defined as a collection of 4,096 features obtained from each *training_data_k_*.

#### RTPFSGA

The motivation to use a genetic algorithm for this approach was to iteratively select distantly related triplet (trimer) pairs. A total of 64 possible triplets were generated and randomly paired during the initialization phase of the genetic algorithm. To build the initial population, we considered a fixed number of random triplet pairs (RTPs) as an individual set of the initial population. Frequencies of each candidate triplet in *RTP_i _*were counted in all promoters and non-promoters in *training_data_k_*; their minimum frequency value was then considered as the frequency of the particular *RTP_i_*. Observing both promoter and non-promoter sequences in each *training_data_k_*, each *RTP_i _*had two frequency values, defined as *X_1 _*and *X_2, _*respectively. For a particular *RTP_i, _*these two frequency values were analyzed by a fitness function, which in turn provided a fitness value for that *RTP_i_*. In the fitness function, a two-tailed student's *t*-test was applied on these two frequency datasets. For this *t*-test we formulated our problem as follows:

• The null hypothesis, μ_0 _: 

• The research hypothesis, μ_a _: 

From the *t*-test, a *t*-value (Eq. 4) was obtained for each *RTP_i_*, which was then used to calculate the density function *f(t) *(Eq. 5), thereby generating the *p*-value (Eq. 6) using the density function.(4)(5)(6)

, where  was the mean of X_1_,  was the mean of X_2_, *t *was the *t*-value from Eq. 4, *abs(t) *was the absolute value of *t*, and *n *was the degree of freedom, which was defined as follows:(7)

, where *n_1 _*was the number of elements in *X_1_*, and *n_2 _*was the number of elements in *X_2_*. The *p*-value was then considered as the fitness value for a particular *RTP_i_*. The assumption was that any *RTP_i _*having a smaller *p*-value than the others has a greater discriminating power. Thus, any *RTP_i _*having a smaller *p*-value was considered as a better fit than the others for the next generation of genetic algorithms, where "Tournament Selection" was used for the survival selection. The best-fit individual between two randomly taken individuals was chosen as the first parent *P_1_*, and the second parent *P_2 _*was chosen in the same way.

Two types of reproduction operators were used in this algorithm: crossover and mutation. The threshold for crossover probability used here was 0.8 and the mutation probability was 0.05. At each step of reproduction, two parent RTPs were checked for crossover. If the probability was less than the threshold, the triplets of both RTPs were swapped with each other. After every crossover action, the mutation probability was checked for every offspring. If the probability was less than the mutation probability, we mutated the offspring. The mutation logic was very simple. First, the part to be mutated was randomly selected, and we then randomly selected a triplet to replace the mutated part. However, we were cautious about the distinct existence of mutated RTPs in the current population. If a mutated RTP was already in the current population, we discarded the choice and search for new mutated part. We generated random double values to simulate these probabilities in order to compare with the corresponding threshold probabilities. The threshold for mutation probability was intentionally set to a relatively smaller value compared to that of crossover so that mutation happens less frequently than crossover.

After the reproduction phase, a fitness value was assigned into each child using the same fitness function (as described above), and two different populations were created: a parent or current population (μ), and a child population (Ω). For the selection of survivors, the (μ + Ω) g → μ mapping approach was used instead of (μ, Ω) → μ, which means that the best-fit individuals (RTPs) in the current population among μ and Ω were selected for the next generation - instead of considering only μ or Ω. Other parameter values of genetic algorithms, except for crossover and mutation probability, were used are as follows: the maximum population size in one generation was 1,000, the number of reproductions in one generation was 500, the maximum child limit in one generation was 500, and the maximum number of generations was 1,000. After tuning several times, these parameter values were fixed (data not shown).

## Results

### Selection of significant features from FDAFSA

**Figure 1:**
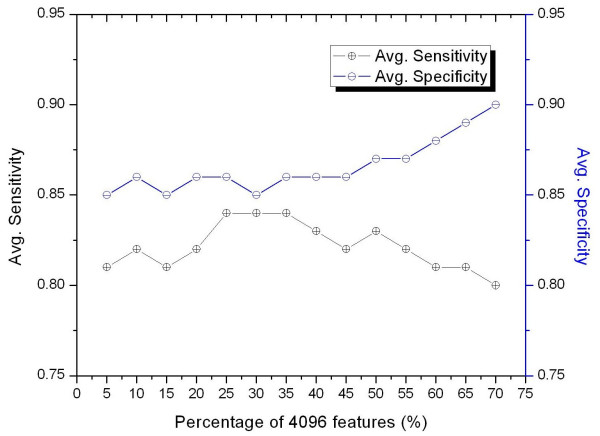
**Average sensitivities and specificities of the FDAFSA method for the selection of a different fraction of features from 4,096 features**. The x-axis shows the fraction of selected features from 4,096 features and the y-axis shows the average sensitivity and specificity corresponding to the selected features.

The accuracy of SVM classification largely depends on the selected features. To select significant features from FDAFSA, we trained our model using a different fraction of features than the *hexamer_set_k _*of *training_data_k _*and tested our model with *test_data_k_*. Figure 1 shows the average sensitivities and specificities of different fractions of 4,096 features. As shown in the figure, the top 25% and 35% feature selections from each *hexamer_set_k _*have the most significant average sensitivity and selectivity at 0.84 and 0.86, respectively. Among these, we selected the top 25% (1,024) features as *hexamer_set'_k _*from each *hexamer_set_k _*rather than the top 35%. The reason for this is that we wanted to keep the size of the feature set as small as possible thus avoiding overfitting. Table 1 presents the top 10 ranked common hexamers from all 5 sets of *hexamer_set'_k_*.

**Table 1:** Top 10 common hexamers in a set of top 25% features of FDAFSA from 5 data sets of 5-fold cross validation.

Rank	Common hexamers extracted from All 5 dataset (top 25%)
1	ATATAT
2	TATATA
3	ATATTT
4	TATAAA
5	AAAAAA
6	TTTTTT
7	AGAGAG
8	TCTCTC
9	CTCTCT
10	GAGAGA

We had chosen hexamers for our analysis because of the empirical results indicating hexamers performing better than the tetramers used in PromMachine [12] (Table 2). We used the same promoter and non-promoter datasets for both methods. For FDAFSA, the average sensitivity and specificity of the 5-fold cross-validation were measured using the top 25% features. We tested the performance of PromMachine using our method. The comparative study revealed that the average sensitivities of these two algorithms were close, though the average specificity of FDAFSA was higher than that of PromMachine.

**Table 2:** FDAFSA vs. PromMachine.

Methods (n-mers used)	Average Sensitivity of 5-fold cross validation (%)	Average Specificity of 5-fold cross validation (%)
FDAFSA (hexamers)	84*	86*
PromMachine (tetramers)	86^+^	81^+^

*Accuracies are measured using the top 25% features from FDAFSA sequences in 1-pass. The measurements are then averaged for 5-passes.

^+ ^This result is generated by implementing the PromMachine algorithm by ourselves using our dataset.

### Selection of significant features from RTPFSGA

**Figure 2:**
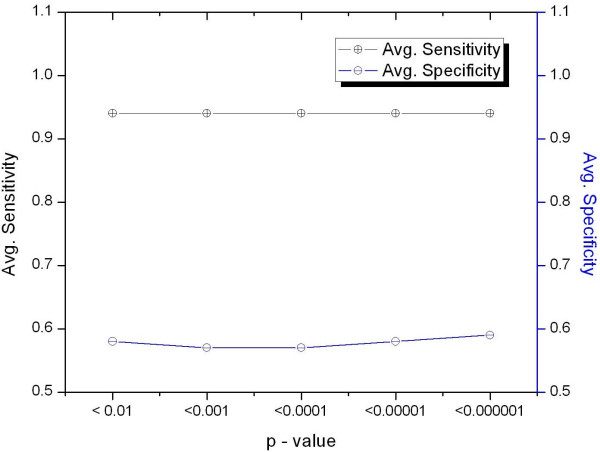
**Average sensitivities and specificities of the RTPFSGA method for different levels of significance (α-value)**. The x-axis shows *p*-values less than the different α-values, and the y-axis shows the average sensitivity and specificity corresponding to the selected features.

After several generations of RTPFSGA, the best-fit RTPs having *p*-value <*α*-value (significance level) were selected for *RTP_set_k _*for each *training_data_k_*. To select the significance level, we trained our model with different *α*-values (0.01, 0.001, 0.0001, 0.00001, and 0.000001) from the *RTP_set_k _*of *training_data_k _*and then tested our model with *test_data_k_*. Figure 2 shows the average sensitivities and specificities for different α-values. The maximum average specificity was 0.59 for α-value of 0.000001, while the average sensitivities for the other α-values were the same as 0.94. Therefore, we selected the features having a *p*-value < 0.000001 and constructed *RTP_set'_k_*. Table 3 shows the 10 most common RTPs for all *RTP_set'_k _*having a *p*-value < 0.000001 using RTPFSGA. The numbers of RTPs in *RTP_set'_a_*, *RTP_set'_b_*, *RTP_set'_c_*, *RTP_set'_d_*, and *RTP_set'_e _*were 161, 200, 173, 167, and 180, respectively.

**Table 3:** 10 common RTPs in a set of RTPs having p-value < 0.000001 of all 5 data sets using 5-fold cross validation.

Rank	Random Triplet Pair (RTP)
1	AAA-AAA
2	AAA-AAT
3	AAA-AGA
4	AAA-ATC
5	AAA-ATT
6	AAA-CAT
7	AAA-TTT
8	AAC-ATA
9	AAC-CGA
10	AAC-CTG

### Combining features

The specificity of FDAFSA was significantly higher than that of RTPFSGA. As shown in Figures 1 and 2, when we chose the top 25% features from FDAFSA, the average specificity of the prediction was 0.86, and the average specificity for features selected by RTPFSGA using a *p*-value < 0.000001 was 0.59. In contrast, the features selected by RTPFSGA had a higher average sensitivity when compared to the sensitivity from FDAFSA (0.94 and 0.84, respectively). Then, in an attempt to increase both the sensitivity and specificity, we merged the two feature sets in PromoBot. For each set of *training_data_k _*we had two feature sets: *hexamer_set'_k _*and *RTP_set'_k_*. We selected only distinct features from these two feature sets to build PromoBot. As RTPs were triplet pairs, two hexamers could be formed from each RTP in *RTP_set'_k_*. In order to construct a unique set of features, the *hexamer_set'_k _*from FDAFSA was checked for the presence of hexamers obtained from RTPs, and these hexamers were subsequently excluded from *hexamer_set'_k_*. Finally, we made *combined_feature_set_k _*from each *training_data_k_*, in which the numbers of features in five combined sets were 1077, 1115, 1096, 1071, and 1097, respectively.

Table 4 shows the prediction result using the combined features. In the table, the average sensitivity was 0.89 and average specificity was 0.86 for promoter prediction using combined features from FDAFSA and RTPSGA, showing an overall enhancement in the classification accuracy. Indeed, the promoter prediction accuracy was significantly increased when using *combined_feature_set_k _*compared to that obtained using features selected by only FDAFSA or RTPFSGA (Table 5).

**Table 4:** Results of prediction test with combined features from FDAFSA and RTPFSGA.

Test Dataset	TP	FN	TN	FP	Sensitivity (%)	Specificity (%)
*test_data_a_*	56	5	52	9	92	85
*test_data_b_*	54	7	52	9	89	85
*test_data_c_*	54	7	55	6	89	90
*test_data_d_*	52	9	51	10	85	84
*test_data_e_*	55	6	51	10	90	84

				Average	89	86

**Table 5:** Comparative accuracy of PromoBot with FDAFSA and RTPFSGA.

Algorithm for feature selection	Average sensitivity for 5-fold cross validation (%)	Average specificity for 5-fold cross validation (%)
FDAFSA	84	86
RTPFSGA	94	59

PromoBot [FDAFSA + RTPFSGA]	89	86

### Comparison with other methods

We compared PromoBot (FDAFSA and RTPFSGA) to other available promoter prediction tools such as Neural Network Promoter Prediction (NNPP) 2.2 [24], Promoter 2.0 Prediction Server [4], TSSP-TCM [6], Promoter Scan 1.7 [25], and PromMachine [12]. For this purpose, the same *training_data k *was used for training PromMachine and PromoBot since the 5-fold cross validation was used for them. For the other tools, the training data was not required. And the same *test_data_k _*was used for testing all the tools. Then, using 5 *test_data_k _*datasets, we measured the sensitivity and the specificity of all tools and then took average of these (Table 6). The comparative assessment showed that NNPP 2.2, TSSP-TCM, and PromMachine had a notable accuracy level, whereas Promoter Scan v1.7 and Promoter 2.0 demonstrated poor predictability. In these tests, PromoBot was found to have a better average sensitivity and specificity than that of NNPP 2.2 (threshold = 0.8). And though there was only a slight improvement in PromoBot's average sensitivity over TSSP-TCM (~1%) and PromMachine (~3%), the average specificity of PromoBot was also marginally better than that of PromMachine (~5%) and TSSP-TCM (2%).

**Table 6:** Comparison with other methods.

Statistical Measure (%)	NNPP 2.2 (threshold = 0.8)	TSSP-TCM	Promoter Scan Version 1.7	Promoter 2.0	Prom-Machine	PromoBot
Avg. Sensitivity	74	88	8	24	86	89
Avg. Specificity	70	84	4	34	81	86

### Performance evaluation using experimentally validated new promoters

In order to evaluate the performance of PromoBot further, we applied the method to a new set of 271 promoters with experimentally validated TSSs. This dataset was downloaded from the recent release (2009.02) of PlantProm database http://linux1.softberry.com/berry.phtml?topic=plantprom&group=data&subgroup=plantprom on January 2^nd^, 2011. Additional File 2 includes information pertaining to gene ID, description, sequence segment location, CDS location, and TSS location for each of these promoters. All sequence segments were from -200 to +51 bp relative to TSS. These new 271 promoters, used as test sequences, did not contain any of the 305 promoter and 305 non-promoter sequences which were used earlier for feature selection and training of PromoBot. We also compared our method with TSSP-TCM. As shown in Table 7, PromoBot accurately classified 235 sequences out of 271 promoters as promoter (86.72% success rate), whereas TSSP-TCM predicted 210 promoter sequences (77.49% success rate). This result confirmed that PromoBot could perform better than TSSP-TCM in detecting promoters.

**Table 7:** Performance evaluation using 271 experimentally validated promoters.

Algorithm	No. of sequences	No. of accurate prediction	Percentage (%)
TSSP-TCM	**271**	210	77.49

PromoBot	**271**	235	86.72

### Comparison of promoter prediction performance using different negative datasets

We also evaluated the effect of using different types of negative datasets on promoter prediction. For this comparison, we collected plant miRNA sequences from miRBase [22] and took 305 sequences having a length greater or equal to 240 bp. Similarly, we collected mRNA and rRNA sequences from PlantGDB[21], selecting 305 sequences from each. In the case of rRNA, we removed sequences having 80% redundancy using Jalview version 2[26] and considered sequences having a length greater or equal to 140 bps.

Using a different type of negative dataset in conjunction with the same positive dataset (the previously used 305 promoters), we extracted features, trained our method, and performed a 5-fold cross validation test in the same way as discussed in the Methods section. Table 8 shows the result of comparative performance analysis between PromoBot and TSSP-TCM when different types of sequences were used as the negative datasets. It should be noted that since TSSP-TCM did not require training data set in order to test whether or not the test sequence is a promoter, TSSP-TCM has same sensitivity value (88%) for all the cases when we tested 305 promoter sequences. But the sensitivities of PromoBot varied because the same positive dataset in combination with different negative dataset were used for feature selection and the 5-fold cross-validation test for each case. The overall performance using rRNA was the best for both algorithms among the sampled ones. The reason for such high performance using rRNA might be due to the presence of redundant information in these sequences. Even though we removed sequences having 80% redundancies, the high degree of conservation of rRNA genes made it impossible to avoid overfitting. Hence, we posit here that it may not be appropriate only to use rRNA as the negative dataset.

**Table 8:** Comparative assessment of performance using different negative datasets

Method	Statistical Measure (%)	miRNA only	mRNA only	rRNA only	PromoBot [miRNA + mRNA + rRNA + tRNA]
PromoBot	Avg. Sensitivity	82.95	87.87	93.12	89
		
	Avg. Specificity	59.67	84.26	95.08	86

TSSP-TCM	Avg. Sensitivity	88	88	88	88
		
	Avg. Specificity	75.41	80.98	96.06	84

In PromoBot--which used a combined negative dataset in which only 40 non-redundant rRNA sequences are included--the overall performance was higher than the case of using only mRNA or miRNA as negative set. The results show effectivity of combining mRNA, rRNA, and miRNA, and tRNA in the construction of the negative set. When only miRNA was used as the negative dataset, the specificities of both programs decreased, though the specificity of TSSP-TCM was significantly better than PromoBot (Table 8). Since discriminating mRNA promoters from miRNA is not an easy task, but an important challenge; further extensive investigations are required for this task. We did not include tRNA sequences for this analysis because there were very few non-redundant tRNA sequences in PlantGDB[21], with considerable variances in sequence length.

## Discussion and conclusions

The comparative improvement of the accuracy rate of promoter predictions by PromoBot indicates that using the frequency distribution of hexamer sequences in combination with RTP analysis can be effective in identifying promoters in plant genomes. This method also has the potential to achieve improved accuracy in promoter identification if extended to genomes of other eukaryotic species.

**Figure 3:**
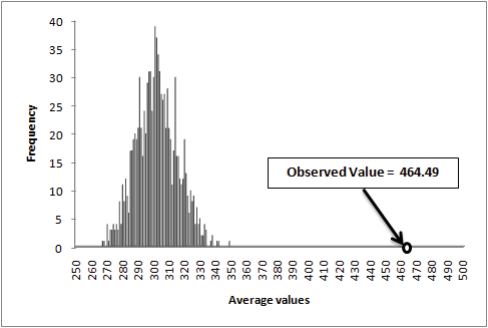
**The significance of RTPs compared to the hexamers produced by two triplets in RTPs**. Observed *diff_RTP_hexamer *average value (464.49) was compared with 1000 random cases where in each case, 220 random triplet pairs were generated and the average of 220 *diff_RTP_hexamer *values was calculated.

In PromoBot, prediction results based on combined features from FDAFSA and RTPFSGA outperformed that based on features extracted from FDAFSA or RTPFSGA alone (Table 5). In order to exhibit how two distantly located triplets in RTPs effectively complemented the hexamers in FDAFSA, we tested the discrimination power of hexamers produced by the concatenation of two triplets in RTPs. For this task, we considered *candidate_hexamer_1 _*to be the concatenation of the first triplet followed by the second triplet in RTP, and *candidate_hexamer_2 _*to be the concatenation of the second triplet followed by the first triplet in the RTP. The discrimination power of the two candidate hexamers (*candidate_hexamer_1 _*and *candidate_hexamer_2_*) could then be measured by the difference of the frequency between promoters and non-promoters. The *diff_RTP_hexamer *in the following equation represents this difference:(8)

, where FD_RTP _was the frequency difference between the RTP in promoters and that in non-promoters, and FD_Hexamer1 _and FD_Hexamer2 _were the frequency differences of two candidate hexamers in promoters and non-promoters for the given RTP, respectively. We found that the discrimination power of two candidate hexamers were smaller, compared to that of RFPFSGA (Additional File 3). Next, *diff_RTP_hexamer *values for 220 RTPs having a *p*-value < 0.000001 from all 305 promoters and non-promoters were calculated, with the average value of 220 RTPs being 464 (Additional File 4). Here, as candidate hexamers, we used the top 1024 hexamers from FDAFSA based on the difference between frequencies in promoters and non-promoters after observing all 305 promoters and non-promoters. In order to show the statistical significance of the observed value of *diff_RTP_hexamer*, we compared the average value of our observed case with the averages of *N *random cases (Additional File 5). For a random case *i*, we randomly generated 220 pairs of triplets, and calculated *diff_RTP_hexamer*. The null hypothesis was that the averages of random cases were greater or equal to the average of our observed case. The *p-value *was calculated using Eq. 9 which is as follows:(9)

, where *N = 1,000*. The average of the observed value (464) had an empirical *p*-value of 0, as shown in Figure 3. Thus, the result confirmed that the RTPs had effectively replaced the weak hexamers and demonstrated their utility as strong features for prediction of plant promoter regions.

Besides using two different algorithms for feature selection, the prediction model in PromoBot has been trained with experimentally identified promoter dataset as well as negative dataset derived from four different sources, i.e. miRNA, tRNA, rRNA and protein coding mRNA genes. With the availability of a large number of plant genome sequences, the accurate identification of promoter regions from such non-coding RNA genes is becoming important. Our analysis showed that the performance of PromoBot varied depending on the negative dataset and that the second highest sensitivity and specificity were achieved when the combination of mRNA, miRNA, rRNA and tRNA gene sequences was used for the negative set (Table 8). Although the use of rRNA alone as the negative data yielded the highest sensitivity and specificity, it might be due to features selected from highly conserved and redundant sequences of rRNA. In the case of the negative dataset consisting of only miRNA genes, the prediction performance was decreased. One of the reasons for this low performance might be the length of miRNA precursor sequences. Plant miRNA precursors are highly variable, with a length ranging from 55-930 bp (average ~146 bp) [27]. Such variation limited our attempt to collect enough miRNA precursor sequences having lengths equal to that of the experimentally verified promoters. Features collected from such sequences might be insufficient for accurate discrimination of RNA pol II plant promoters from miRNA genes. Also, miRNA genes may have other strong features that are unrecognized by the FDAFSA and RTPFSGA in PromoBot. In the future, statistical and biological features of miRNA genes will be studied in detail to fully utilize these features for improvement of prediction algorithm.

Recently, a hierarchical stochastic language algorithm that utilizes the analysis of hexamer occurrence frequencies in DNA sequences has been shown to be successful in accurately recognizing transcriptional regulatory regions in several species including Arabidopsis and rice [28]. This usefulness of hexamers in identifying promoter sequences is also confirmed by our results (Table 5), demonstrating high sensitivity and specificity (84% and 86%, respectively) in case of FDAFSA. Also, the utilization of RTP alone in discriminating promoter and non-promoter datasets resulted in highly improved sensitivity (94%) in the test datasets. However, unlike hexamers, use of RTP information did not yield high specificity. This may be due to several reasons. First, the protein coding sequences in the training dataset were obtained from multiple species. While this approach is useful for avoiding species specificity in the prediction method, it also means that there was no specific codon usage bias present in the collected protein sequences. Also, our non-promoter dataset contained protein-coding sequences and other non-coding gene sequences such as tRNA and miRNA; such diversity may have caused noise in the RTP analysis and it is quite possible that the RTP analysis may have shown more specificity for non-promoter sequences if the coding sequences were taken from a single species. Nevertheless, we assumed from the results that RTPs may also have some other significance in the promoter regions of the genome, as it was found that the DNA curvature of promoters is higher than that of coding regions [9]. Thus, distal elements may become proximal to the core promoter elements and contribute to the regulation of gene expression. However, a more detailed study is required in order to explore and identify the significance of RTPs in promoter regions in greater detail.

## Competing interests

The authors declare that they have no competing interests.

## Authors' contributions

AKMA developed and implemented a method to predict plant promoters and wrote the manuscript. SS helped in collecting data sets and helped in writing the manuscript. NN initiated and directed this research. HL directed the research and helped in writing the manuscript. All authors read and approved the final manuscript.

## Supplementary Material

Additional file 1 **List of plant species**. List of plant species from where mRNA, tRNA, rRNA, and miRNA selected as non-promoter sequences. The number of each type of RNA sequences is also included. Click here for file

Additional file 2 **New set of 271 experimentally validated promoters**. Sequence details of 271 experimentally validated promoters. Information of gene ID, description, sequence segment location, CDS location, and TSS location are included. Click here for file

Additional file 3 **Comparative performance analysis of RTPFSGA with FDAFSA with respect to feature frequency**. Frequency analysis of 220 RTP having a p-value < 0.000001 and a frequency analysis of corresponding candidate hexamers found in 1,024 hexamers (from FDAFSA). Click here for file

Additional file 4 **Distribution of frequency for 1,000 random RTP cases**. Distribution of frequency for 1,000 random cases. Click here for file

Additional file 5 **Frequency analysis of the observed RTPs**. Frequency analysis that demonstrates the differential discriminating power between a particular RTPs and two corresponding candidate hexamers. Click here for file
